# Evaluation of measurement reliability for selected indices of postural stability based on data from the GYKO Inertial Sensor System

**DOI:** 10.5114/biolsport.2024.132986

**Published:** 2023-11-17

**Authors:** Janusz Jaworski, Grzegorz Lech, Kazimierz Witkowski, Rafał Kubacki, Paweł Piepiora

**Affiliations:** 1 Faculty of Physical Education and Sport, University of Physical Education in Kraków, Poland; 2 Faculty of Physical Education and Sports, Wroclaw University of Health and Sport Sciences, 51-612 Wrocław, Poland

**Keywords:** Postural stability, Accelerometer, Test-retest reliability, Intraclass correlation coefficient, Students

## Abstract

The main aim of this study is to use comprehensive statistical analyses to evaluate measurement reliability of selected variables that characterize postural stability. The study examined twenty-nine healthy non-athlete students. The examinations were performed twice, with a one-week interval. The Microgate GYKO inertial sensor system was used to evaluate the reliability of variables that characterize postural stability. The relative reliability of the repeated test was evaluated using the intraclass correlation coefficient (ICC) with 95% confidence interval (95% CI). Next, the standard error of measurement (SEM) and minimal detectable change (MDC) were computed. Relative reliability of the repeated test for all analysed variables of ICC ranged from 0.31 to 0.75. For four variables, ICC values were ca. 0.7, i.e., they can be considered as good. For four other variables, ICC ranged from 0.41 to 0.54, with these values considered fair. Satisfactory reproducibility of postural stability measurements using the GYKO inertial sensor system demonstrates that it can offer an inexpensive and efficient alternative to measurements that use force balance platforms.

## INTRODUCTION

In many sports (e.g., skiing, swimming, team games), balance is one of the leading coordination (neurophysiological) abilities. Therefore, monitoring the ability to maintain balance is critical for the comprehensive analysis of the level of athletes’ motor preparation, and during recruitment and selection for teams [1, 2]. A high level of postural stability is also needed to perform everyday activities in a safe and independent manner and to reduce the risk of falling [3, 4].

Data from various types of balance platforms are most often used to measure various indicators of postural stability. The results obtained provide a benchmark for other studies (“gold standards”). They allow for evaluation of postural stability based on displacement of the COP (centre of pressure) on the supporting surface during relaxed standing (standing with both feet and one foot) [4, 5]. Unfortunately, these platforms are usually very big and expensive, while their installation and software are complex [6]. Another group of equipment for balance measurements is Wii Balance Boards [7, 8, 9]. In population studies, balance is examined by means of various motor coordination tests. Among them, the most popular is the Flamingo Balance test from the Eurofit Physical Fitness Test Battery [10]. Other tests have also been used for many years: the Stork Balance Stand Test, the Y Balance Test, the Stick Lengthwise Balance Test, the Balance Beam Test [11]. Due to the aforementioned limitations of posturographic platforms, postural stability is measured by means of various types of accelerometers. They are cheaper, more mobile, and easy to use in population studies [12, 13, 14]. The use of accelerometery [15, 16, 17, 18, 19] for recording body sway gained popularity when the costs of accelerometers with improved measurement parameters fell, and wireless technology became commonplace. ICC (intraclass correlation coefficient) values obtained during postural stability measurements performed by means of accelerometers are good, usually over 0.75 [20]. Studies have also demonstrated a strong correlation between results obtained from force platforms and accelerometer data. The location of accelerometers on the human body is also an important issue due to various links between the movements of individual body segments and COM (centre of mass) displacements used to maintain postural stability. In his research, Golema, based on correlation coefficients, determined the relationships between COM and body trunk displacements. The results demonstrated that body trunk displacements (displacements of a point located near the body trunk centre of gravity) can be considered the same as those of COM. This assumption applies only to maintaining postural stability in undisturbed conditions [21].

Therefore, in our study, we measured the variables that characterize postural stability by means of the GYKO inertial sensor system (Microgate, Bolzano, Italy) located on the body trunk of study participants.

The primary objective of this report was to evaluate the repeatability of measurements of selected variables characterizing postural stability.

## MATERIALS AND METHODS

### Study group

The study examined 29 healthy non-athlete students (age = 21.67 ± 0.88 years, body weight = 179.82 ± 6.87 cm, body mass = 75.55 ± 7.62 kg). The tests and anthropometric measurements were performed in accordance with the Declaration of Helsinki. The examinations were approved by the Bioethics Committee at the Regional Medical Chamber in Kraków, Poland (approval No. 159/KBL/OIL/2017). The tests were conducted within statutory research No. 106/BS/IS/2016.

### Technical specifications of the GYKO device

GYKO is an inertial measurement unit (IMU), consisting of an accelerometer for recording the following variables: power and speed of lifting a weight and monitoring of postural stability (recording of several variables). It offers options for analysis of body sway during gait, running and jumping, and evaluation of postural stability. A detailed description of the technical specifications of the accelerometer can be found in the manual [22].

### Testing protocol

The participants performed all the tests barefoot, in the same room, between 8 a.m. and 3 p.m. A test was performed for 30 s while standing on both feet, without moving, with eyes open and arms relaxed along the sides of the body. Feet were positioned hip width apart and the participant was asked to look at a point on the wall at a distance of ca. 2.5 m. The group of university students always performed the first and second series of examinations at the same time of the day. The same research team supervised both test series. The tests and retests were conducted with a one-week interval. This approach has often been used in studies examining test reliability [23, 24]. During the first examination, we determined the height at which the GYKO system was to be attached. According to the manufacturer’s recommendations, this height should be set at the level of the T1-T2 thoracic vertebrae. During the second examination, the GYKO system was attached again at the same height. The tension of the GYKO attaching straps (chest circumference) was also adjusted to each student. The wireless transmission protocol was used to transfer data recorded by the GYKO inertial sensor to the laptop.

### Scope of the study

We examined the following variables characterising postural stability [25, 26, 27]: area [mm^2^], convex hull area [mm^2^], length (ML) [mm], length (AP) [mm], mean distance (ML) [mm], mean distance (AP) [mm], RMS distance (ML), RMS distance [mm], (AP) [mm], mean velocity (ML) [mm/s], mean velocity (AP) [mm/s].

### Statistical analysis

1. The Shapiro-Wilk test was used to assess variables for normal distribution, whereas Levene’s test was employed to assess the equality of variances.
2. Basic descriptive statistics (arithmetic mean, standard deviation) were computed for all the variables in both series of examinations (test, retest) and for the material in total.
3. The relative reliability of the repeated test [28, 29] was evaluated using the intraclass correlation coefficient (ICC) with 95% confidence interval (95% CI).
4. Apart from ICC, we also computed standard error of measurement (SEM), which is used to express absolute measurement reliability [29], according to the formula:
  $$\text{SEM}=\text{SD}\sqrt{1-\text{ICC}}$$
  where:
  SEM – standard error of measurement
  SD – standard deviation from test 1 and 2
  ICC – intraclass correlation coefficient
5. Next, SEM% values were computed as
  SEM%=(SEM/grand mean)×100
  This method was used to express SEM as a percentage of the mean value from the test and retest for each variable to allow comparison of absolute reliability between the measurements obtained in various units [30, 31, 32, 33].
6. SEM was used to evaluate the minimal detectable change (MDC), interpreted as the lowest value of changes needed to exceed the error of measurement for two repeated tests at specific CI. Computations were performed for 95% CI using the formula:
  $${\text{MDC}}_{95}=\text{SEM}\times 1.96\times \sqrt{2}$$
7. Next, MDC_95_ was expressed as the percentage of the mean from the test and retest, according to the formula:
  $${\text{MDC}}_{95\%}=({\text{MDC}}_{95}/\text{grand mean})\times 100$$
8. The differences between means from the test and retest used the repeated-measures Student’s t-test. We used the non-parametric Wilcoxon pair test if the distribution of variables differed from normal. Furthermore, the Cohen effect was also computed for each variable [34].

The calculations were performed using STATISTICA software (v. 12, StatSoft, Krakow, Poland), and Excel (2016 MSO, Microsoft Corporation, Redmond, WA, USA). Furthermore, the effect size was determined by means of GPower 3.1 free software and available for Mac OS X and Windows, which is widely used in social studies [35].

## RESULTS

Table 1 illustrates basic statistical characteristics for test and retest variables. They were used for computation of the standard error of measurement (SEM), expressed as a percentage mean from test and retest (SEM%) and minimal detectable change (MDC_95%_).

**TABLE 1:** Basic statistical characteristics of the analysed postural stability indices

Variable [unit of measurement]	(Test + Retest)/2 ± SD	Test ± SD	Retest ± SD
Area [mm^2^]	839.48 ± 380.63	824.83 ± 394.33	854.13 ± 372.81
Convex hull area [mm^2^]	581.46 ± 309.36	611.92 ± 352.61	555.01 ± 262.11
Length (AP) [mm]	266.34 ± 51.03	276.78 ± 49.62	255.90 ± 51.12
Length (ML) [mm]	192.02 ± 42.16	196.60 ± 42.51	187.44 ± 42.06
Mean distance (AP) [mm]	9.17 ± 3.67	8.83 ± 3.77	9.50 ± 3.62
Mean distance (ML) [mm]	4.45 ± 1.88	4.73 ± 2.12	4.16 ± 1.60
RMS distance (AP) [mm]	11.30 ± 4.54	10.84 ± 4.85	11.76 ± .,25
RMS distance (ML) [mm]	5.57 ± 2.35	5.92 ± 2.67	5.21 ± 1.98
Mean velocity (AP) [mm/s]	8.80 ± 1.69	9.14 ± 1.64	8.45 ± 1.69
Mean velocity (ML) [mm/s]	6.34 ± 1.39	6.49 ± 1.40	6.19 ± 1.39

As mentioned in the Introduction section, the aim of this report was to evaluate reliability indices for selected variables that characterize postural stability. The values of intraclass correlation coefficient (ICC), standard error of measurement (SEM) and minimal detectable change (MDC_95_) are presented in Table 2. Relative reliability of the repeated test for all analysed ICC variables ranged from 0.31 to 0.75. The lowest ICC values were obtained for mean frequency (AP), whereas the highest values were found for area. For four variables, ICC values were ca. 0.7, i.e., they can be considered as good to excellent. Furthermore, for four variables, ICC values ranged from 0.41 to 0.54 and can be considered fair. Furthermore, low ICC values were obtained for two variables: mean distance (AP) and RMS distance (AP). It should be emphasized that all low ICC values concerned anterior-posterior sway (AP).

**TABLE 2:** Values of intraclass correlation coefficient (ICC), standard error of measurement (SEM) and minimal detectable change (MDC_95_) for the analysed indices of postural stability.

Variable [unit of measurement]	ICC	ICC (95% CI)	SEM	SEM%	MDC_95_	MDC_95%_
Area [mm^2^]	0.75	0.53÷0.88	190.31	22.66	527.51	62.83
Convex hull area [mm^2^]	0.68	0.42÷0.84	176.66	30.29	481.36	82.85
Length (AP) [mm]	0.46	0.11÷0.71	37.49	14.07	103.91	39.01
Length (ML) [mm]	0.70	0.45÷0.85	23.09	12.02	64.00	33.32
Mean distance (AP) [mm]	0.31	-0.06÷0.61	1.56	17.01	4.32	47.10
Mean distance (ML) [mm]	0.54	0.22÷0.76	1.27	28.53	3.52	79.10
RMS distance (AP) [mm]	0.35	-0.02÷0.63	1.89	16.72	5.23	46.28
RMS distance (ML) [mm]	0.41	0.05÷0.67	1.75	31.41	4.85	87.07
Mean velocity (AP) [mm/s]	0.46	0.11÷0.71	1.24	14.09	3.43	38.97
Mean velocity (ML) [mm/s]	0.70	0.45÷0.85	0.76	11.98	2.10	33.12

ICC – coefficient of intraclass correlation, 95% CI – confidence interval, SEM – standard error of measurement, SEM% – percentage standard error of measurement, MDC – minimal detectable change.

Table 2 also presents standard error of measurement (SEM), which is used to express absolute measurement reliability. In general, SEM% for all analysed variables ranged from 11 to 30%. High ICC = 0.70 values at low standard error of ca. 11–12% were obtained for length (ML) and mean velocity (ML). Furthermore, the highest SEM% was found for convex hull area, which was also characterized by the highest measurement reliability.

The SEM values were used to compute the minimal detectable change, MDC_95_. For the analysed variables, the values ranged from 33% to 82%. The lowest values of MDC_95%_ were obtained for length (ML) and mean velocity (ML). The highest MDC_95%_ was again obtained for convex hull area. In general, analysis of the data shown in Table 2 concerning MDC_95_ and ICC revealed high values at good and satisfactory measurement reliability.

The study also analysed the statistical significance of differences between the results of individual variables obtained for test and retest (Table 3) and Cohen effect size. Statistically significant differences were found only for two variables: length (AP) and mean velocity (AP). It should be emphasized that the likelihood, p = 0.040, was close to the limit of the set level of statistical significance (p ≤ 0.05). No statistically significant differences were found between other variables. As suggested by Cohen, the effect size for six variables was small (below 0.2), whereas a medium effect was observed for the remaining four variables (between 0.22 and 0.30).

**TABLE 3:** Results of the evaluation of the significance of differences in the analysed variables of postural stability between test and retest and Cohen’s effect sizes.

Variable [unit of measurement]			
	t (W)	p	C^d^
Area [mm^2^]	-0.577729	0.568	0.056908
Convex hull area [mm^2^]	0.746482	0.122	0.192026
Length (AP) [mm]	2.142798	0.040*	0.308932
Length (ML) [mm]	1.501903	0.144	0.161460
Mean distance (AP) [mm]	0.659508^W^	0.509	0.235702
Mean distance (ML) [mm]	1.654175^W^	0.098	0.309534
RMS distance (AP) [mm]	0.789247^W^	0.429	0.176544
RMS distance (ML) [mm]	1.469692	0.152	0.224612
Mean velocity (AP) [mm/s]	2.145012	0.040*	0.150311
Mean velocity (ML) [mm/s]	1.503076	0.144	0.160290

Without marking – value of the Student t-statistic for dependent samples; W – value of the Wilcoxon test statistic; C – Cohen’s effect size, d > 0.2 = small, > 0.5 = medium, > 0.8 = large.

* Significant at p ≤ 0.05.

## DISCUSSION

The reliability of a diagnostic method is one of the most important factors that determine its usefulness. Determination of the reliability and validity of measurements with respect to the so-called gold standard has practical implications for athletes and coaches. The main aim of this study was to verify measurement reproducibility for selected variables that characterize postural stability using comprehensive statistical analyses. A mobile Microgate GYKO inertial sensor system was used in the study. We will refer the results of our study to only certain comparative materials. This methodological approach was adopted due to the high number of scientific studies on reliability of postural stability measurements (various measurement apparatus, tests during standing on one leg, two legs, on stable or unstable ground, with eyes open or closed, with hands on hips or crossed on the chest, tests for the population of healthy people and those with geriatric problems, tests of people of different ages).

There are no unequivocal criteria for the evaluation of measurement reliability in the literature. Landis and Koch [36] proposed adopting the following reliability criteria: 0.01 to 0.20 – slight; 0.21 to 0.40 – fair; 0.41 to 0.60 – moderate; 0.61 to 0.80 – substantial; 0.81 to 1.00 – almost perfect. These reliability ranges have been respected by many researchers, but they have also been rejected or modified by others. The following criteria for ICC have also been adopted in studies: values in the range of 0.80 to 1.00 are considered excellent reliability, values in the range of 0.60 to 0.80 represent good reliability, while an ICC lower than 0.60 means poor reliability [30, 31]. Munro proposed a popular classification of ICC: values between 0.00 to 0.25 reflect little correlation; 0.26 to 0.49 – low correlation; 0.50 to 0.69 – moderate correlation; 0.70 to 0.89 – high correlation; and 0.90 to 1.00 – very high correlation [37]. Given various methodological approaches to balance tests, the classification proposed by Fleiss et al. [38] used in many publications seems to be the most justified, with ICC values > 0.75 labelled excellent, > 0.40 – fair to good, and < 0.40 – poor. This interpretation of ICC was also adopted in our study. Analysis of the literature concerning this subject reveals that in comparative studies, ICCs ranged from average and poor to high [24, 39]. The results obtained in our study for ICC reliability coefficients support these findings. Most of the reliability indices calculated in our study (according to the adopted classification) can be considered as fair and high. The ICC values were below 0.40 (poor) only for two variables i.e., mean distance (AP) and RMS distance (AP). In general, slightly lower values of ICC in our study were observed compared to the studies that used platform tests (ICC = 0.46–0.98 [40, 41] and Wii Balance Boards (ICC = 0.64–0.91 [7, 42, 43]. ICC reliability results of postural stability measurements obtained with different types of accelerometers are good and are usually around 0.7 [44, 45, 46]. Similar ICC values (from 0.62 to 0.70) to our own study, for the test performed on the dominant limb, were obtained by Jaworski et al. [47]. The GYKO accelerometer was also used in the cited study.

The interpretation of ICC values does not provide much insight into the actual changes in the variables that could be used for practical interpretation. Consequently, many authors [29, 31, 32] suggest that absolute values of measurement reliability should also be interpreted. For this purpose, it is necessary to compute SEM and MDC to enhance identification of the levels of errors for the analysed variables. It should be emphasized that with large interindividual variability, high values of ICC can be obtained even if the absolute reliability is low. Evaluation of the test result interpretation error is directly related to its reliability. This means that the higher the SEM is, the lower are the absolute test reliability and accuracy of the results [30]. In our study, SEM% for all most variables ranged from 10 to 20%. This reflects good absolute measurement reliability. SEM values were also used to compute the minimal detectable change (MDC). This index is defined as the minimum value of changes needed to distinguish the actual change from the change caused by the variability of results or errors of measurement [48]. Therefore, evaluation of MDC for each variable that characterizes postural stability provides information about the errors of measurements that should be taken into consideration during determination of the smallest important changes expected for two consecutive measurements [49]. We obtained relatively high MDC values at good and fair measurement reliability.

### Limitation of the study

The authors are aware of the limitations of this report. Therefore, in future research, we propose to increase the size of the research group, and to conduct the tests in different chronological age groups of both sexes, attaching the GYKO device in the lumbar region of the spine (L3).

## CONCLUSIONS

1. Values of the intraclass correlation coefficient (ICC) for the variables of postural stability should be considered as good and fair.
2. High reproducibility of postural stability measurements using the GYKO inertial sensor system demonstrates that it can offer an inexpensive and efficient alternative to measurements that use force balance platforms.

## Data Availability

The data used to support the findings of this study are available from the corresponding author upon request.
